# Advanced pancreatic ductal adenocarcinoma with liver metastases treated with multimodal therapy: a case report

**DOI:** 10.3389/fonc.2026.1745057

**Published:** 2026-03-18

**Authors:** Lei Cao, Bin Liu, Jinheng Liu, Hongwei Wu, Mulan Zhong

**Affiliations:** 1 Interventional Oncology, Chengdu Qingbaijiang District People’s Hospital, Chengdu, China; 2 Sichuan Provincial Hospital of Integrated Traditional Chinese and Western Medicine, Chengdu, China

**Keywords:** case report, liver metastases, multidisciplinary team, pancreatic ductal adenocarcinoma, therapy

## Abstract

**Introduction:**

To investigate the therapeutic efficacy and survival benefits of a multidisciplinary team (MDT) strategy, including local interventional therapy, systemic chemotherapy, and immunotherapy, for patients with advanced pancreatic ductal adenocarcinoma (PDAC) presenting with liver metastasis at initial diagnosis.

**Case presentation:**

A 54-year-old male patient was diagnosed with PDAC in the tail of the pancreas with multiple intrahepatic metastases (cT3N1M1, stage IV). The patient underwent multimodal comprehensive treatment, including iodine-125 seed implantation at the pancreatic primary site, eight cycles of hepatic arterial infusion chemotherapy, PD-L1 inhibitor (Benmelstobart), and oral S-1 maintenance therapy. After treatment, the patient’s liver metastases significantly decreased in size (partial response according to RECIST 1.1), CA19–9 levels dropped from 4141.57 U/mL to 58.64 U/mL, overall survival exceeded 15 months, and quality of life remained good (ECOG score 1).

**Conclusion:**

The MDT strategy, particularly the combination of local interventional therapy with systemic treatment and immunotherapy, can effectively control both the primary and metastatic lesions of advanced PDAC, significantly prolonging patient survival and providing valuable treatment references for similar cases.

## Introduction

Pancreatic ductal adenocarcinoma (PDAC) is one of the most aggressive solid tumors. Approximately 80% of PDAC patients are diagnosed with locally advanced or distant metastasis, with a median survival of less than 12 months and a 5-year survival rate of less than 10% (1). For metastatic PDAC patients, the prognosis is even worse, with a median survival of less than 6 months and a 5-year survival rate of less than 1% (2). Although traditional chemotherapy regimens (such as FOLFIRINOX and gemcitabine plus nab-paclitaxel) can improve the prognosis of some patients to a certain extent, the overall efficacy is still limited, and drug resistance often occurs (3).

In recent years, with the continuous development of interventional therapy, immunotherapy, and molecular targeted drugs, the multidisciplinary comprehensive treatment (MDT) strategy for PDAC has gradually gained attention. For example, radioactive seed implantation can enhance local control of the primary tumor (4); hepatic arterial infusion chemotherapy (HAIC) can significantly increase the drug concentration in liver metastases (5); and immune checkpoint inhibitors (such as PD-1/PD-L1 inhibitors) can enhance antitumor immune responses by modulating the tumor microenvironment (6). However, how to integrate these treatment modalities into an efficient and safe individualized treatment plan and ultimately extend the survival of patients with advanced PDAC remains a major challenge in current clinical practice.

This article reports a case of advanced PDAC with liver metastasis at initial diagnosis, who achieved long-term survival through a multimodal comprehensive treatment strategy, including iodine-125 seed implantation, HAIC, PD-L1 inhibitors, and oral S-1. This case aims to explore the potential value of MDT in advanced PDAC and provide useful references for the clinical management of such patients.

## Case presentation

**Figure 1:**
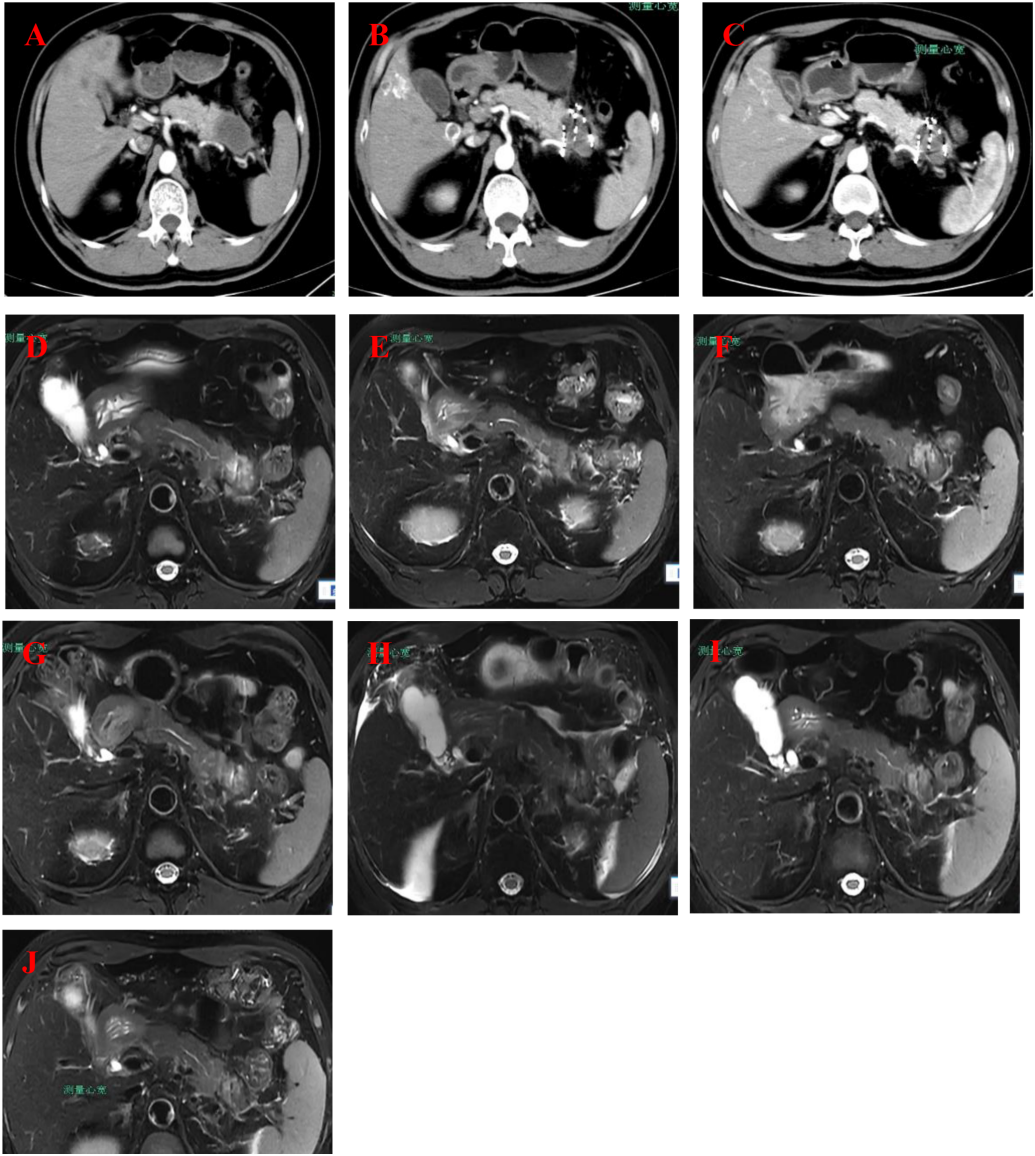
Medical imaging progress of pancreatic and liver conditions. **(A)** (2024-4-4): CT scan of the abdomen showing pancreatic tail mass, suspected as a malignant tumor, with liver metastasis and enlarged lymph nodes. **(B)** (2024-5-13): MRI scan of the abdomen depicting a pancreatic tail tumor, with signs of liver metastasis, gallbladder inflammation, and possible kidney involvement. **(C)** (2024-6-11): CT and MRI showing unchanged pancreatic mass with metastatic liver lesions, mild gallbladder inflammation, and lymphadenopathy. **(D)** (2024-8-21): MRI scan highlighting the pancreatic mass, possible splenic vein thrombosis, and multiple liver lesions showing no significant change. **(E)** (2024-10-9): MRI of the abdomen showing a stable pancreatic mass with continued liver metastasis and a possible inflammatory process in the kidneys. **(F)** (2024-11-25): MRI scan showing pancreatic tumor with minimal changes, continued liver metastases, and moderate kidney abnormalities. **(G)** (2024-12-16): MRI showing unchanged pancreatic tumor, suspected splenic vein involvement, and stable liver lesions with mild gallbladder inflammation. **(H)** (2025-1-13): MRI showing slight growth in pancreatic tumor, stable liver metastases, and increased abdominal fluid accumulation. **(I)** (2025-2-5): MRI depicting stable pancreatic mass, unchanged liver metastases, and decreased pleural fluid. **(J)** (2025-2-26): MRI showing slight reduction in the edge enhancement of the pancreatic tumor, with stable liver metastases and mild pleural effusion.

**Figure 2:**
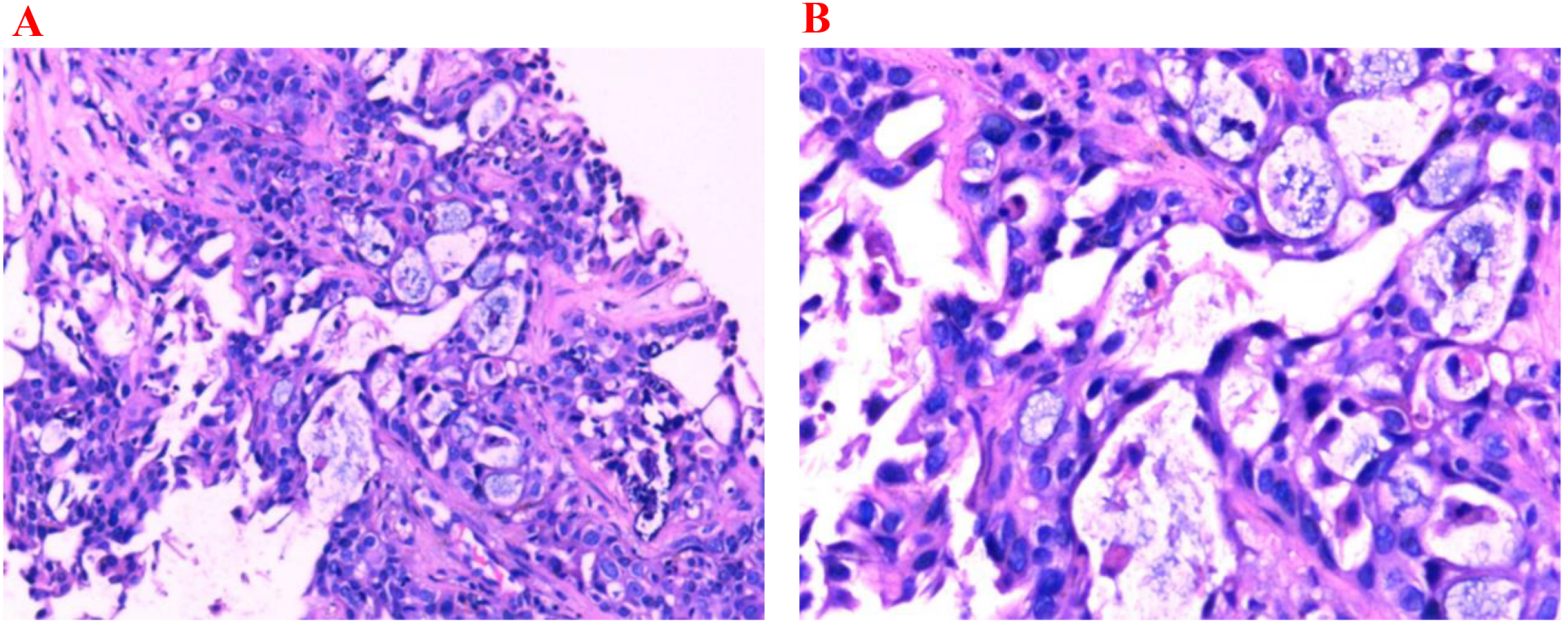
Histopathological images stained with hematoxylin and eosin (H&E). **(A)** (H&E ×100) shows deeply stained nuclei (high chromatin) with variable size and shape (pleomorphism). **(B)** (H&E ×200) reveals cells arranged in glandular patterns, with some areas showing dense cell clusters forming solid cords or irregular glandular lumens; nuclei are significantly enlarged with an increased nuclear-to-cytoplasmic ratio; cytoplasm is moderate to scanty and eosinophilic; cell arrangement is disordered with partial loss of polarity.

**Figure 3:**
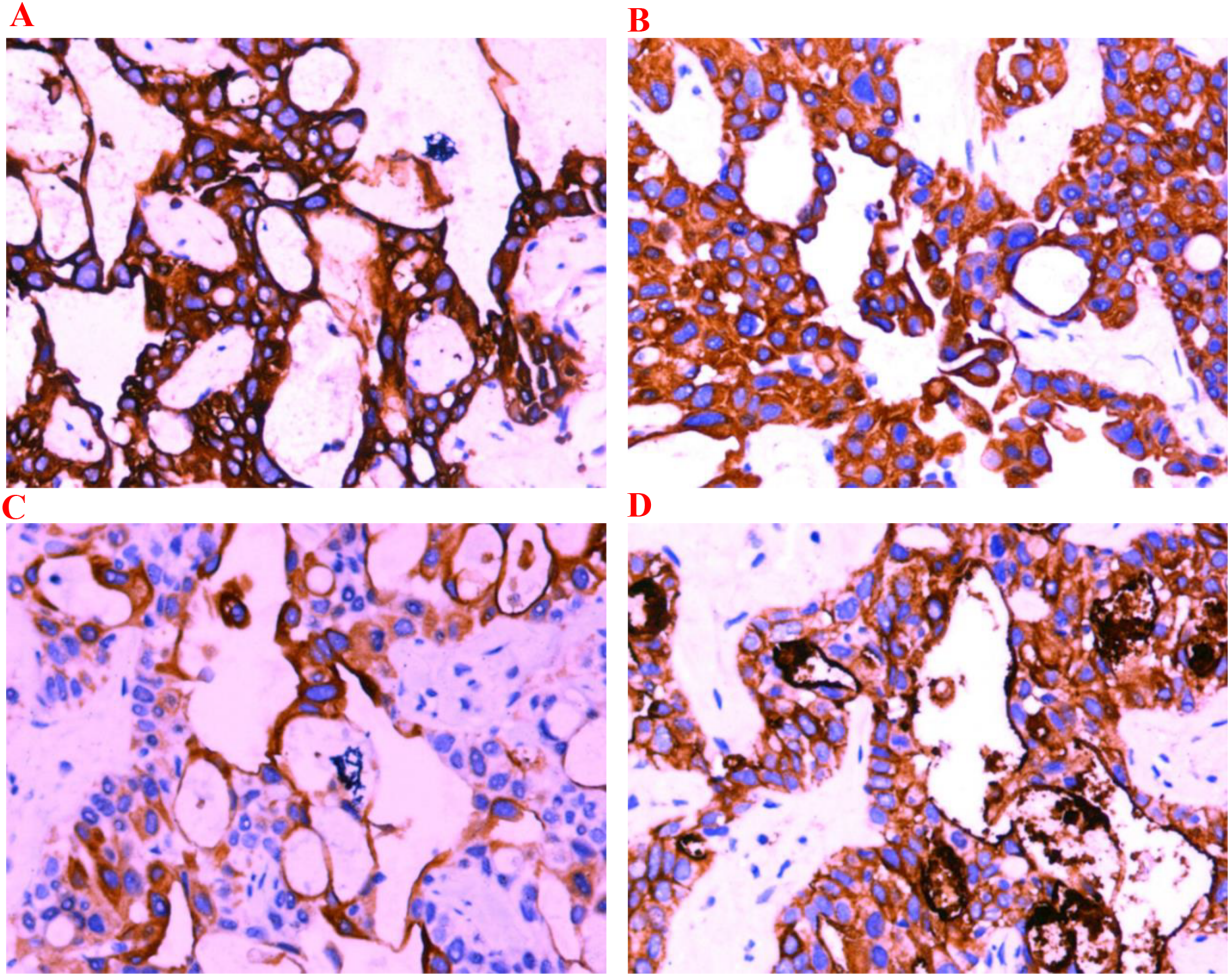
Immunohistochemistry (×200). **(A)** CK7 positive; **(B)** CK19 positive;**(C)** CK20 positive; **(D)** CA125 positive.

The patient is a 54-year-old male who was admitted to the hospital on April 4, 2024, due to a “pancreatic mass detected during a physical examination.” He had a history of hypertension for more than 10 years, well controlled with irbesartan and amlodipine besylate, with no other significant past medical history, and no known family history of malignancy. Enhanced CT scan of the whole abdomen revealed a mass in the tail of the pancreas (approximately 4.9 cm × 3.0 cm × 2.7 cm) with multiple liver metastases and enlarged peripancreatic lymph nodes (**Figure 1A**), with no signs of other distant metastases. Laboratory tests on April 15, 2024, showed a significant elevation in serum CA19–9 levels, reaching 4141.57 U/mL. Pathological results from ultrasound-guided liver mass biopsy confirmed pancreatic ductal adenocarcinoma (**Figure 2**). Immunohistochemical tests on April 16, 2024, showed positive results for CK7, CK19, CK20, and CA125 (**Figure 3**). The patient was clinically staged as cT3N1M1 (stage IV).

**Figure 4:**
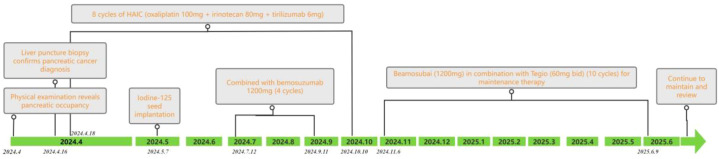
Timeline of treatments and investigations.

From April 2024 to February 2025, the patient underwent multidisciplinary comprehensive treatment, with the specific treatment plan and timeline shown in **Table 1**, **Figure 1**, and **Figure 4**.

**Table 1:** Clinical timeline and therapeutic response in pancreatic cancer with liver metastases: CA19–9 monitoring and interventional management (2024–2025).

Date	Examination/treatment item	Key findings/procedure details	Clinical significance/treatment response	CA199 (U/mL)
2024-04-04	Abdominal contrast-enhanced CT, Brain MRI	- Pancreatic tail mass (suspected malignancy); Multiple liver metastases; Enlarged lymph nodes in the pancreatic head region - Brain MRI: Bilateral frontal lobe ischemic foci	Initial findings suggestive of pancreatic cancer with liver metastases	–
2024-04-15	Laboratory tests	Significant elevation of CA199	Tumor activity marker	4141.57
2024-04-18	Hepatic arteriography + drug perfusion (Cycle 1)	First interventional therapy	Good postoperative recovery, preliminary control of liver metastases	–
2024-05-07	Iodine-125 seed implantation (external hospital)	Local radiotherapy intervention	Local control of the primary lesion	–
2024-05-13	Upper abdominal contrast-enhanced CT/MRI, Lab tests	- CT: Post-particle implantation changes; Shrinking liver metastases; Abnormal enhancement in left kidney (metastasis to be ruled out) - MRI: Consistent with pancreatic cancer with liver metastases - CA199 reduction	Partial remission post interventional therapy + particle implantation	2806.72
2024-05-16	Hepatic artery perfusion (Cycle 2)	Second interventional therapy	Continued control of liver metastases	–
2024-06-11	Upper abdominal contrast-enhanced CT/MRI, Lab tests	- CT: Progressive shrinkage of liver metastases; Irregular left kidney contour - MRI: Post-interventional changes in liver metastases (partial shrinkage) - Significant CA199 decline	Marked therapeutic response	709.67
2024-06-14	Hepatic artery perfusion (Cycle 3)	Third interventional therapy	Maintenance of therapeutic efficacy	–
2024-07-11	Hepatic artery perfusion (Cycle 4)	Fourth interventional therapy	–	–
2024-07-12	Bemotuzumab immunotherapy (Cycle 1)	Initiation of maintenance immunotherapy	Enhanced systemic control through combination therapy	–
2024-07-30	Laboratory tests	CA199 approaching normal range	Stable disease status	78.67
2024-08-01	Hepatic artery perfusion (Cycle 5)	Fifth interventional therapy	–	–
2024-08-21	Upper abdominal contrast-enhanced MRI	- Stable pancreatic lesion size - Splenic vein involvement/thrombosis (collateral circulation formation) - Widening of partial metastases in the left medial liver lobe	First indication of splenic vein complications	–
2024-08-22	Hepatic artery perfusion (Cycle 6)	Sixth interventional therapy	–	–
2024-09-12	Hepatic artery perfusion (Cycle 7)	Seventh interventional therapy	–	–
2024-10-09	Upper abdominal contrast-enhanced MRI, Lab tests	- Splenic vein collateral circulation formation - Stable left kidney lesion - CA199 is maintained at a low level	Long-term stability	56.81
2024-10-10	Hepatic artery perfusion (Cycle 8)	Eighth interventional therapy	Completion of interventional therapy cycles	–
2024-11-06	Bemotuzumab immunotherapy (Cycle 2)	Continuation of immunotherapy	–	–
2024-11-25	Upper abdominal contrast-enhanced MRI	- Slight shrinkage of liver metastases - Minimal ascites	Local progression (ascites suggests potential peritoneal metastasis)	58.65
2024-12-16	Upper abdominal contrast-enhanced MRI	- Stable pancreatic lesion - Mildly increased ascites	Requires monitoring of fluid changes	57.16
2025-01-13	Upper abdominal contrast-enhanced MRI	- Slight enlargement of pancreatic tumor - Significant increase in ascites - Bilateral pleural effusion	Disease progression (primary lesion enlargement + systemic fluid accumulation)	58.90
2025-02-05	Upper abdominal contrast-enhanced MRI	- Reduced pleural effusion after absorption - Stable remaining lesions	Improvement following supportive care (diuretics + anti-infection)	57.30
2025-02-26	Upper abdominal contrast-enhanced MRI, Lab tests	- Reduced marginal enhancement of pancreatic tumor - Stable splenic vein thrombosis - CA199 is maintained at a low level	Possible reduction in tumor activity	58.64

### Local interventional therapy

In May 2024, the patient underwent iodine-125 seed implantation at the pancreatic primary site. Follow-up imaging showed stable size of the primary lesion with dense deposition of multiple seeds within.

### Systemic treatment

The patient completed eight cycles of hepatic arterial infusion chemotherapy (HAIC) (oxaliplatin 100 mg + irinotecan 80 mg + tirilizumab 6 mg), combined with Benmelstobart (1200 mg per dose, intravenous infusion, infusion time 60 min per dose, every 3 weeks for a total of four cycles). Subsequently, the patient entered the maintenance treatment phase, with a regimen of Benmelstobart combined with oral S-1 (60 mg per dose, twice daily, continuous administration for 21 days followed by a 14-day break, for a total of 10 cycles).

### Supportive care

On December 18, 2024, the patient received anti-tumor therapy. On December 30, 2024, he experienced grade III myelosuppression due to chemotherapy, with decreased white blood cells and platelets, and was managed with subcutaneous granulocyte colony-stimulating factor (G-CSF) and interleukin-11. The patient received further anti-tumor therapy on February 26, 2025. Subsequently, the patient experienced a second episode of grade III myelosuppression on March 19, 2025, which was managed with supportive care including G-CSF, pegylated recombinant human G-CSF, and interleukin-11 subcutaneously (**Table 2**).

**Table 2:** Treatment-related adverse events and corresponding interventions.

Date	Event/management
2024-12-18	Received anti-tumor treatment
2024-12-30	Experienced grade III myelosuppression; administered 150 μg of human granulocyte colony-stimulating factor and 3 mg of interleukin-11
2024-12-31–2025-1-8	Received 300 μg of granulocyte colony-stimulating factor and 3 mg of interleukin-11
2025-1-9–2025-1-14	Received 300 μg of granulocyte colony-stimulating factor
2025-2-26	Received anti-tumor treatment
2025-3-19	Experienced grade III myelosuppression
2025-3-19–2025-3-21	Received 300 μg of granulocyte colony-stimulating factor
2025-3-22	Received 6 mg of pegylated recombinant human granulocyte colony-stimulating factor
2025-3-25–2025-3-26	Received 3 mg of interleukin-11

**Figure 5:**
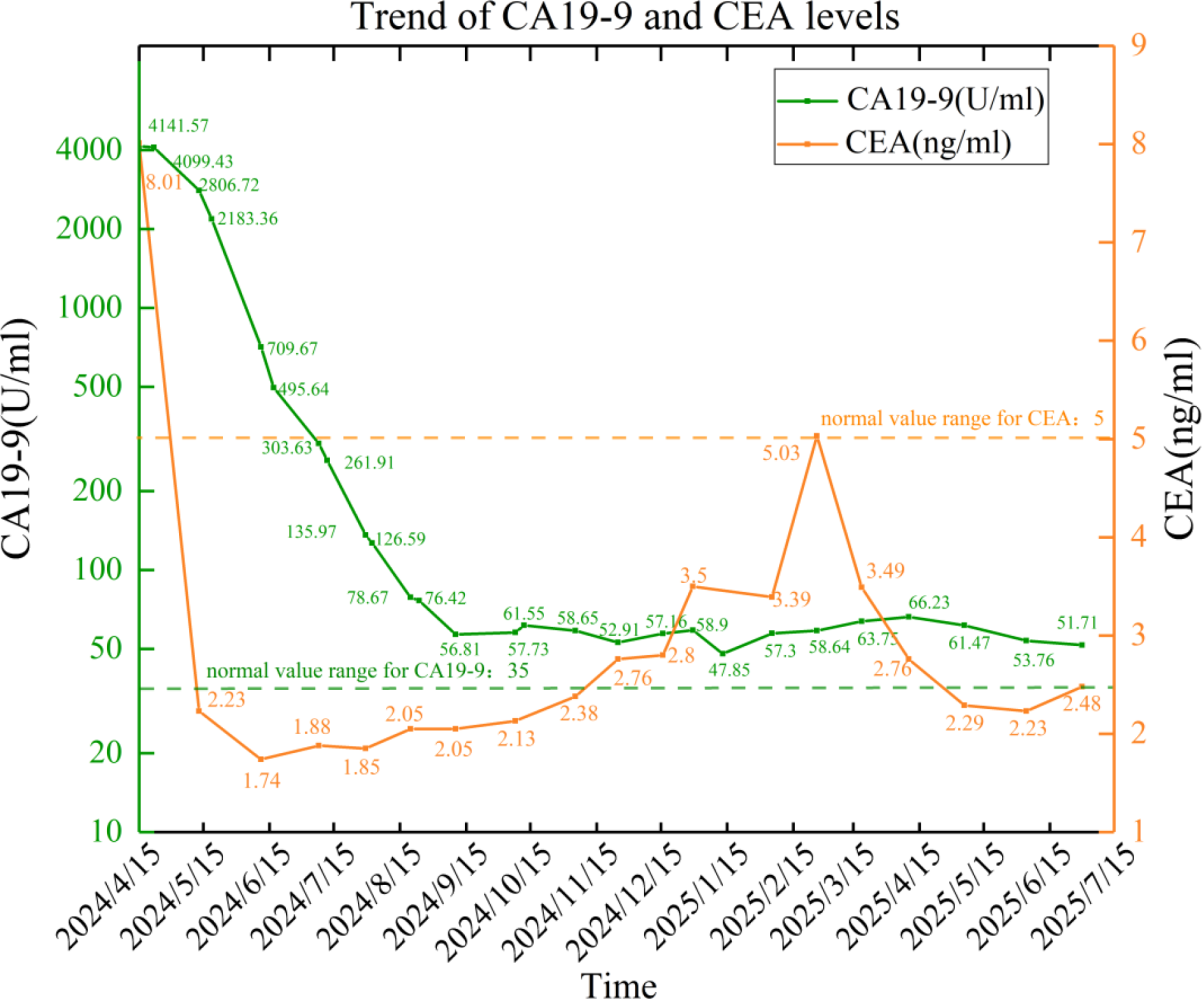
Trend of CA19–9 levels (U/ml) and CEA (ng/ml) levels.

During follow-up, the patient’s tumor markers significantly improved. CA19–9 levels decreased from the baseline of 4141.57 U/mL to 58.64 U/mL in June 2025, while CEA remained within the normal range. The dynamic changes are shown in **Figure 5**. Imaging assessment revealed a 45% reduction in the sum of the longest diameters of liver metastases (partial response according to RECIST 1.1 criteria), with the pancreatic primary lesion remaining stable (**Figure 1B**). As of July 2025, the patient’s overall survival had reached 15 months, with an ECOG performance status of 1 and no signs of disease progression.

## Discussion

This case describes an advanced PDAC patient who achieved an overall survival of 15 months following a combination treatment strategy involving iodine-125 seed implantation, HAIC, PD-L1 inhibitors, and S-1. Currently, standard systemic therapy for metastatic PDAC primarily comprises FOLFIRINOX or gemcitabine-based regimens, with reported median overall survival ranging from 6.7 to 11.1 months. By comparison, at the time of data cutoff, the patient in this case had achieved an overall survival exceeding 15 months. At the most recent follow-up, the overall survival was approximately 21 months, accompanied by a favorable quality of life. These findings suggest that a multimodal treatment strategy may offer additional clinical benefit in selected patients. The individualized therapeutic approach was applied in real-world clinical practice and may provide an important reference for the multidisciplinary comprehensive treatment of advanced PDAC. The analysis is now unfolded from three dimensions: synergistic mechanisms of treatment, technological innovation and safety control, and dynamic monitoring and precision treatment.

In this patient, a sequential therapeutic strategy was implemented, beginning with HAIC to control liver metastases, followed by iodine-125 seed implantation for sustained local tumor control, and subsequently combined with immunotherapy and maintenance therapy. Iodine-125 seed implantation, as a form of precision radiotherapy, may exert anti-tumor effects through dual mechanisms. On one hand, its continuous low-dose radiation can potentially induce double-strand breaks in tumor cell DNA within the effective range. In this case, the primary lesion remained stable for 11 months after the procedure, which is broadly consistent with the reported local control rate of 85.71% in the literature (7). On the other hand, seed implantation may also have a radiosensitizing effect. Studies suggest that irradiation at 6 Gy combined with 30 nM gemcitabine can significantly upregulate the Bax/Caspase-3 pathway, increasing the apoptosis rate of PANC-1 cells to 41.2% (8). HAIC may additionally enhance drug delivery to liver metastases through a “first-pass effect,” with floxuridine exposure in the liver reaching 6–8 times that of systemic chemotherapy (9). In this case, a 45% reduction in liver metastases was observed, which roughly aligns with the 66.1% objective response rate (ORR) in the combination treatment group. The sequential treatment model of HAIC → seed implantation → PD-L1 inhibitor could potentially stimulate anti-tumor immune responses through a series of chain reactions, including promoting antigen release, enhancing antigen presentation, and T-cell recognition.

In terms of technological application and safety, the 3D-printed coplanar template technique significantly enhances the precision of seed implantation. It achieves a target V100 of 91.17% while keeping the duodenal irradiation dose below 45 Gy (10), effectively avoiding severe complications. In this case, there were no occurrences of pancreatic fistula or perforation, which is consistent with reports that the template technique reduces the incidence of severe complications to less than 5% (11). HAIC also demonstrates advantages in terms of safety. Studies have shown that the mFOLFOX regimen can reduce total bilirubin levels by 46.56% in patients with hyperbilirubinemia (12), with hematological toxicity primarily consisting of grade 1–2 events (12). HAIC also demonstrated acceptable safety in this patient, enabling continuation of subsequent systemic therapy over a relatively long course.

From the perspective of dynamic monitoring and treatment guidance, the 98.6% reduction in CA19–9 levels in this case is consistent with the characteristics of a treatment-sensitive population (patients with a reduction of >50% within 3 months have an OS of 14.2 months) (13). A comprehensive multidimensional monitoring system should also include parameters from multiple categories such as inflammation-nutrition indicators, molecular features, and radiomics. For example, patients with serum albumin ≥3.5 g/dL and CRP <0.3 mg/dL have a 62% lower risk of disease progression (14); patients with a KRAS mutation abundance reduction of over 30% have a 3.5-fold increase in response rate to S-1 therapy (15). The dynamic changes in the CT values of the primary lesion in this case also reflect the biological effects of the continuous radiation from the seeds, which is associated with dosimetric features of V100>90%. These observations indicate that integrated dynamic monitoring may help inform treatment adjustment and response evaluation in patients undergoing multimodal therapy.

As a single case report, these findings do not replace evidence from prospective clinical trials but indicate the potential feasibility of a multidisciplinary, multimodal treatment strategy in selected patients with advanced PDAC. The observed outcomes suggest that a tripartite treatment framework integrating “local control, systemic clearance, and immune activation” should be further developed. It is recommended that future research focus on the following areas: First, the development of a dynamic prognostic model incorporating ctDNA clearance rate to achieve more precise efficacy assessment; second, in-depth exploration of the mechanism by which S-1 enhances the immune therapeutic effect by downregulating COL1A1; and third, optimization of the spatiotemporal combination strategy of seed implantation and HAIC to enhance treatment synergy. This case underscores the potential value of a multidisciplinary, multimodal approach in advanced PDAC and may serve as a practical reference for developing individualized treatment plans in the future.

## Conclusion

This case, through a multidisciplinary comprehensive treatment strategy, including the combination of iodine-125 seed implantation, HAIC, PD-L1 inhibitors, and S-1, enabled a patient with advanced PDAC and liver metastasis to achieve an overall survival of more than 15 months, demonstrating good clinical benefits.

## Data Availability

The original contributions presented in the study are included in the article/supplementary material. Further inquiries can be directed to the corresponding author.
